# The high-performance sport environment: laying the foundation for a new research topic

**DOI:** 10.3389/fspor.2025.1503199

**Published:** 2025-03-11

**Authors:** Anusofia Schlawe, Ask Vest Christiansen, Kristoffer Henriksen

**Affiliations:** ^1^ Institute of Sport Science and Clinical Biomechanics, University of Southern Denmark, Odense, Denmark; ^2^ Department of Public Health, Aarhus University, Aarhus, Denmark

**Keywords:** sport psychology, elite sport, high-performance environment, wellbeing, organisational culture

## Abstract

Talent development and dual career literature have drawn attention to the importance of the environment in athlete development and highlighted that certain types of environments are more successful at supporting athletes to develop and perform. No such literature exists at the elite level, and it remains unclear how high-performance sport environments (HPSEs) can foster both the current and future capacities of athletes while simultaneously striving to support their wellbeing. The aim of this paper is therefore, to provide the foundation required for this question to be explored. Through the integration of five pivotal discourses—athlete career development, applied elite sport psychology, the holistic ecological approach in sport psychology, athlete mental health, and elite sport policy—this paper provides a conceptual definition of the HPSE and a typology of environments. While no research has yet made the HPSE the central object of investigation, many adjacent lines of research point to factors that may be characteristics of HPSEs that support performance and wellbeing, including: a holistic approach that balances performance objectives with athletedevelopment and wellbeing, a facilitative organisational culture, and a personalised and caring coaching philosophy. This paper has implications for stakeholders and key staff working in HPSEs who are interested in improving the organisation of their environment and the wellbeing and performance of their athletes

## Introduction

Elite athletes do not reach the top on their own. To realise their dreams of international sporting success, athletes rely on a network of key support structures including coaches, managers, technical experts, family, and friends. Moreover, the path to success is reliant upon key institutions, for example, various non-government organisations and national Olympic committees. In 1996, Hardy and colleagues asserted that “elite athletes do not live in a vacuum; they function within a highly complex social and organisational environment, which exerts major influences on them and their performances” (1). Nevertheless, unlike at the talent development level, where landmark research has demonstrated the role of the environment in an athlete's successful development (2) research has yet to examine the unique social and organisational environments within which elite athletes are embedded, and to understand how these environments support athletic performance and wellbeing.

The overarching goal in elite sport is to achieve high-level performance. However, the traditional “win-at-all-costs” approach that has historically dominated elite sport cultures has been increasingly challenged, particularly in light of growing discussions surrounding elite athlete mental health. This discourse has been driven by evidence of destructive high-performance sport environments (HPSEs) in the United States (3), Scandinavia (4) and the United Kingdom (5). As a result, there has been a shift toward considering how we might cultivate environments that uphold a core performance focus while also prioritizing athlete mental health. This shift is reflected in several Position Stands (6–9), as well as broader initiatives within professional sports, such as collaborative efforts by sports unions to promote mental health awareness and governing bodies launching campaigns aimed at safeguarding individuals in elite sport (10–12). While attention to such approaches in elite sport has grown—illustrated by research examining the development of psychologically informed performance contexts (13, 14) — these environments remain aspirational rather than the norm. Consequently, a significant gap in the literature persists regarding how to effectively cultivate them. This paper seeks to begin the process of filling this gap by conceptualising and contextualising the concept of the HPSE, and by mapping current research related to the elite environment. In doing this, we hope to lay a foundation upon which further research can build.

## Positioning of this paper

There has been a growing demand for conceptual papers in academia (15) the absence of which has been suggested to create a loss of breadth and depth in academic thought and empirical research (16). The present paper is a conceptual paper that is inspired by the theory synthesis paper that seeks to provide a contextually grounded, theoretical conceptualization and lay the early foundations for future research within a new field. Studies of elite sport and their contexts are not situated within one established research field and remain fragmented across diverse theoretical perspectives, conceptual frameworks, and ontological and epistemological positions, a systematic review of the literature was not considered viable. Instead, the present paper draws upon elements of a narrative review which allows unexplored research topics to be explored through open-ended research questions (17).

A theory synthesis paper affords the possibility for new patterns and connections to be uncovered, offering new lenses through which to explore a given phenomenon (18). Drawing on ideas from five relevant discourses, we aim to provide a preliminary understanding of (1) what a HPSE is (2) the different types of HPSEs that exist (3) the features that may characterise good environments with the goal of providing a foundation for future research that can explore the contextual nuances of each type of environment and the success factors associated with each.

## Contextualising the high-performance sport environment

While the Athletic Talent Development Environment (ATDE) has been extensively studied, primarily through two dominant frameworks (19), research related to high-performance sport has been approached from multiple perspectives and with varying purposes, rather than a single, unified position. As a result, there is no overarching definition that clearly defines and delineates what constitutes a HPSE. While there can be overlaps, the context of elite sport is markedly different from that of talent development; an ATDE's primary purpose is to help prospective elite athletes make a successful transition from the junior to senior elite level, and to serve as a talent pipeline**,** nurturing a wide base of developing athletes—recognising that not all will ultimately compete at the elite level. By contrast, the primary purpose of an HPSE is sustaining or enhancing peak performance among established athletes. This emphasis on immediate performance often results in funding and investment being closely tied to athletes' success. Moreover, elite athletes typically experience greater involvement from media and sponsors, and possess higher earning potential compared to those competing at the developmental level (15). These characteristics reinforce the need for specialised research on HPSEs, as emphasised by Henriksen and Stambulova (16).

The elite sport landscape is highly diverse, and each HPSE is unique, shaped by the specific demands of the sport it serves and the broader context in which it operates. Consequently, we use the term HPSE as a broad conceptual anchor to define a context that, despite variations in structure and resources, shares a common emphasis on optimising athlete performance and addressing fundamental needs related to peak performance. We acknowledge that an HPSE within a professional club governed by a league may differ from those operating under the purview of an Olympic Training Centre. However, we propose that HPSEs can be effectively examined through shared principles—such as comprehensive performance support services, a performance-driven focus, and the fulfillment of basic psychological needs—which are broadly relevant across elite sport contexts. Despite their differences, HPSEs typically share structural elements that endure across various environments.

At the helm of HPSEs are individuals or teams responsible for managing the environment, such as boards of directors, shareholders, or management teams**.** These entities bear the primary responsibility for shaping the environment's vision and overarching strategy. A Director of Performance or Performance Manager is typically tasked with overseeing the daily operations, implementing performance strategies, and ensuring effective communication and collaboration among staff (20). HPSEs tend to have robust expert support teams where athletes have contact with a diverse range of experts and support personnel (21). This can include full-time professional physiologists, biomechanists, psychologists, nutritionists, physiotherapists, medical doctors, data scientists, strength and conditioning coaches, tactical coaches, performance coaches, head coaches, and input from external advisors who possess relevant expertise (e.g., academic advisors) (18, 20). The focus on short term performance success (e.g., season to season) typically favoured in high-performance sport often results in frequent changes to the staffing and performance team (22). This transience appears to be unique to HPSEs and can present a challenge in creating an enduring organisational culture and structure.

## Discourses to conceptualise the high-performance sport environment

We suggest that future research into HPSEs as a phenomenon can be situated across five key discourses: the athlete career discourse, the holistic ecological approach in sport psychology, the athlete mental health discourse, the elite sport policy discourse, the applied elite sport psychology discourse. A discourse is understood as a historically constructed and shared body of knowledge on a given topic (23). The selected discourses were chosen because they provide relevant perspectives that collectively contribute towards a holistic understanding of HPSEs. While distinct in focus, they overlap in their relevance to high-performance sport, reflecting the complex and dynamic nature of HPSEs.

## The athlete career discourse in sport psychology

The athlete career discourse encompasses research on career development, dual careers, career transitions, and career assistance, illustrating the evolution of thought on the athletic career. Current perspectives favour a holistic lifespan approach (24) that emphasises a whole-person, whole-career, and whole-environment orientation, where an athletic career is not only a stage of life but also a foundation for lifelong development. This perspective has influenced how we conceptualise success in HPSEs in this paper, framing it through the concept of career excellence. Career excellence (23) transcends traditional performance metrics and is defined as an athlete's ability to sustain a healthy, successful, and long-lasting career in sport. Health in this context reflects adaptability and resourcefulness, while success is understood as the pursuit of meaningful goals both within and beyond sport. This conceptualisation aligns with a holistic lifespan perspective and serves as a benchmark for evaluating HPSEs and as a goal for them to strive toward (24).

## Holistic ecological approach in sport psychology

 The Holistic Ecological Approach (25) has been instrumental in talent development research, shifting focus from an athlete-centred view of talent to a contextually situated understanding of development. Rooted in Bronfenbrenner's (26) work on the ecology of development, this discourse emphasizes that development results from a complex interaction between the individual, their environment, and the broader sociocultural context over time. The HEA highlights the tangible processes and factors that enable optimal development performance and wellbeing in talented young athletes and views an ATDE as a dynamic system that operates across micro and macro levels (27). The micro level encapsulates an athlete's immediate surroundings and the interrelations between these surroundings. It is at the micro level that athletic and personal development take place. The macro-level reflects the larger context within which these surroundings are embedded and the organisational culture of the sports club or team.

Research in talent development has clearly illustrated the influence that nourishing environments have on positive athlete development and has demonstrated that the ATDEs which have been successful in supporting athlete wellbeing and long-term development share common characteristics. These include: a whole person approach, an integration of efforts, role models, support for athlete autonomy (19). Although not directly embedded with HEA research, a recent focus on the twilight zone between the individual and the policy level has clearly demonstrated the importance of aspects such as organisational culture, leadership, and the organisation of environments.

## Athlete mental health discourse

The athlete mental health discourse has gained increasing attention in recent years (6, 27), as mental health is now recognized as a fundamental component of sustained high performance (7). While elite athletes experience psychological distress at rates comparable to the general population, they also face unique stressors associated with high-performance sport (28). These include competition pressure, exhaustive training demands, balancing athletic and non-athletic commitments, career transitions, underperformance, and the psychological effects of overtraining and injury (29). It is now generally agreed that supporting athlete mental health should be a component of HPSEs, and that athlete mental health must considered in addition to performance (6). However, the role of the environment itself in shaping mental health remains underexplored, and further research is needed to understand how staff and organizational structures collectively contribute to athlete well-being.

## Elite sport policy discourse

 Elite sport policy discourse has focused on understanding how macro structures and national policy factors influence a nation's sporting success. The impact of sport policy factors on international sporting success has been examined in several seminal works (27, 30). This research has identified key factors that support successful elite sport systems across nations, and focused on understanding the relationships between population, wealth, geography, and sport systems and policies and long-term performance. Macro factors fundamental for effective elite sport systems include the provision of appropriate facilities, coaching and sport science services, adequate funding and financial support, and sufficient high-quality competition and competition opportunities (31). This research has been widely used to shape national elite sport frameworks across the world.

## The applied elite sport psychology discourse

 Elite sport demands a commitment and investment beyond that typically required in other career paths; elite athletes have little time to cultivate social networks outside of their sport; they have little time to devote to a parallel career and as a result, are financially dependent on being successful in their sport; they may struggle to develop a non-sporting identity, and may find that their self-concept and self-worth are dependent on their success as an athlete. These factors can be catalysts for stress and can affect performance and overall wellbeing if athletes do not learn to manage their responses to external pressures (32). The applied sport psychology discourse centres on understanding the psychological determinants of expert performance and developing athletes who are resilient to the pressures of elite sport in addition to the stressors that arise in everyday life.

Sport psychologists take different intervention approaches, such as psychological skills training (33) cognitive behavioural therapy (34) existential psychology (35) and acceptance commitment therapy (32) but all agree that mental fortitude is a key to success and can be developed.

 The role of the sport psychologist is evolving. Research has illustrated that issues related to organisational functioning can be a distinguishing factor in achieving Olympic success, and that the modern sport psychologist is required to play a key role in mediating organisational conflicts and developing the organisational culture (36). Eubank and colleagues (22) illustrate that sport psychologists assist with managing conflicts between coaches and athletes, communication among staff and athletes, financial matters, media scrutiny, and obligations to sponsors and team ownership. Sports psychologists can help athletes navigate these challenges not only on an individual level, but also on an organisational level by working to foster a facilitative and psychologically safe culture (37). In this way, sport psychologists are now expected to be cultural architects and competent mediators, in addition to experts in performance psychology.

## Bridging the discourses to investigate high-performance sport environments

To research and develop sustainable and successful HPSEs, it is necessary to draw on all five discourses. By situating future research within these five discourses, we aim to provide a framework for understanding HPSEs that speaks to both individual and systemic influences. While these discourses overlap in certain areas, they each offer distinct perspectives relevant for understanding the HPSE. In acknowledging these overlaps, we highlight the need for integrated approaches that examine HPSEs holistically, incorporating insights across research disciplines.

The holistic ecological discourse draws our attention to the HPSE as a dynamic system with components, a structure, and functions. The elite sport policy discourse supports this idea and directs attention to policy factors to provide a macro level context for the specific HPSE. The athlete career discourse and the concept of career excellence illustrates that the HPSE marks a unique and distinct phase of the athlete journey and helps to conceptualise the successful HPSE as supporting athletes in their sporting and personal lives, encouraging holistic development, and demonstrating a long-term commitment to their athletes' development. The athlete mental health discourse further develops the concept of a successful HPSE as one that promotes athlete thriving. Finally, the applied elite sport psychology discourse reminds us that sport psychology is an applied discipline and that researching HPSE should lead to actionable strategies that improve the environments of athletes. Taken together, these discourses provide a foundation for research into HPSEs.

## The High-performance sport environment: working definition and typology

 There exists no conceptual clarity on how to define an HPSE. We therefore draw on key themes from the five relevant discourses to define an HPSE as:

a dynamic system comprising (a) at the micro level an elite athlete's immediate sport team and their non-sport daily surroundings, (b) the organisational culture and leadership of the sports team (c) at the macro-level, the larger context, including national sport policy and funding, in which the team is embedded.

The elite sport landscape is highly diverse, and athletes will interact with a variety of different environments across their athletic career. The nature of these environments differs depending on the broader sporting and national contexts. These differences influence the way that environments are managed, structured, and operated, and their approach to supporting athletes' wellbeing and performance. There is a need to consider the different types of HPSEs that exist, with a view to understanding what are unique and common characteristics. Inspired by Morris and colleagues' (38) taxonomy of dual career development environments, the following non-exhaustive taxonomy of HPSEs classifies different types of environments and outlines key features of each and the broader context within which they are situated. 

## The professional team environment

Professional sports team environments operate primarily as business entities with financial goals, evident in popular sports such as football, basketball, and team handball. The success of athletes in these arenas translates to monetary benefits that extend beyond the individual, benefiting a network of stakeholders including the clubs, broadcasting entities, sponsors, agents, managerial personnel, and investors. These environments are largely financed by investment through “ownership”, which can create a tension between profit generation and egalitarian sporting values. Sponsorship significantly influences the dynamics of these environments—both the individual athletes and the overarching club structure. Athletes often find themselves navigating dual allegiances, for instance balancing commitments to their personal sponsors against those pledged to the club (39).

Typically, athletes' salaries are the greatest expense for the club or team, and athletes can earn substantial salaries (40). Such environments are characterised by continual flux and a culture of uncertainty; athletes may be loaned out, bought or sold in an arena which tends to be international and transient. This extends to the management staff, who are typically hired on short-term contracts, where the opportunity for extension depends almost entirely on continued performance success. The unique funding model of professional environments means they typically have more money to support state-of-the-art facilities, a full suite of experts and support staff (20).

## The national team gathering environment

A national team gathering environment consists of players loaned from professional clubs with the purpose of competing in select national competitions. Such environments are formed for short periods throughout a competitive season and are often created for specific competitions (e.g., world cups). These environments typically exist in sports that traditionally have strong professional clubs (e.g., football, ice hockey, basketball). It is common for athletes in these environments to have infrequent contact outside of the competition period, and many may play for professional clubs that compete against each other in professional leagues. These environments face unique challenges, in that athletes are brought together for short periods where stakes are typically very high. This creates a context where environments exist for a specific purpose, where national pride is often the dominant driver over financial compensation. Additionally, these environments predominantly operate under the purview of national sports policy frameworks, which may diverge from the operational policies of the professional clubs with which athletes are usually affiliated.

## National and olympic training centres

Academy training environments are those that exist under a central coordinating organisation responsible for Olympic and Paralympic Sport. These environments are designed to improve conditions for the development of elite sport across various disciplines. Examples can be found in Norway, Australia, Great Britain, and the United States, which all have centralised Olympic training centres. These centres are fully equipped for athletes and offer housing, dining, recreational facilities, in-house sports medicine and sports science services (41). Unlike professional clubs, these environments are not commercial enterprises, and while they may have sponsors, these environments are not subject to pressure from sponsorship or stakeholders. Similarly, these environments are national centres, and therefore only support athletes from their respective nations, making them less international and more heterogenous.

##  The national team environment 

The national team environment is one in which athletes from the same sport have regular, ongoing commitments, and where the national team is the primary setup. These environments are designed to support athletes in their daily training, and therefore athletes have access to a range of support and coaching staff. Typically, sports that are organised within national team frameworks are those that lack sufficiently large national professional leagues. Such environments are funded predominantly by national sporting bodies and supported by sponsors. Examples can be found in Danish badminton, archery and sailing.

## The club environment

 The club environment reflects a training and competition environment that supports elite athletes as well as semi-elite and often junior athletes. This creates a unique dynamic where elite athletes often find themselves training alongside their less-experienced counterparts, which may foster a sense of mentorship and comradery. Governance in these settings is typically vested in a central federation, which mandates standards, best practices, and finances for all affiliated clubs. A hallmark of club environments is their relative stability, contrasting with the more transitory nature of other sports settings. This stability typically engenders a sense of community and belonging among athletes and creates a connection and commitment to the club as a unique entity. Given that a club environment is not as encompassing as other elite sport settings, it often provides greater flexibility for elite athletes which may enable them to maintain a balanced life beyond their athletic commitments. A club environment may have a professional department and thus be a mix of an amateur club and a professional club environment.

This typology, with its five different environments, is not exclusive. Individual athletes may have unique environments; a professional tennis player may be surrounded by a team of their choosing and is not typically embedded within a routine daily training environment. Notwithstanding, this typology highlights the dynamic and varied nature of the elite sport landscape; these environments share performance aims, yet are structured differently, and are beholden to unique demands from stakeholders. This reinforces the need for a targeted investigation of HPSEs, using contextually sensitive approaches to understand the unique similarities and differences.

## Key aspects of high-performance sport environments

On the one hand, no research project to date has made HPSEs the central object of investigation, nor has any study set out to holistically describe an HPSE or systematically structure the factors that may explain its success. Some studies have examined environments that cater to both young talented athletes and elite athletes (26, 41) or dual career development environments (DCDEs) that included elite level athletes (42). However, these studies conceptualized such environments as ATDEs or DCDEs rather than HPSEs, and the existing body of research remains too limited to constitute a comprehensive review.

On the other hand, research on specific and often isolated factors contributing to success in high-performance sport is abundant. These factors span psychological domains such as decision-making and mental skills, social dimensions such as leadership and group dynamics, physiological aspects such as nutrition and training methodologies, and technological innovations. However, these factors are highly diverse, context-dependent, and often examined in isolation, making it difficult to consolidate them within a single, unified model of HPSEs.

Rather than imposing an overarching framework that forces all elements into a single structure, we recognize that HPSEs are complex, dynamic, and contextually distinct environments. In the following, drawing from a narrative review approach (17), we highlight key factors that have been emphasised in the literature as relevant considerations for future research on HPSEs. While some principles from existing approaches may provide useful insights, we acknowledge that HPSEs require a distinct conceptualisation, and further empirical investigation is needed to refine this understanding.

Given proven track record of the Environment Success Factors (ESF) model in structuring factors that contribute to the success of ATDEs, we draw on this model to provide a structure for the review of literature findings as they relate to the HPSE.  We organise themes according to the categories comprising the model: preconditions, processes, and organisational culture. Preconditions refer to characteristics of an environment that are a prerequisite for elite performance but do not alone guarantee success. Processes reflect actions, behaviours and occurrences that happen repeatedly and are central to the daily functioning of the environment. Individual development reflects the personal development of individual athletes while team development reflects development related to the group, or environment as a whole. Organisational culture refers to the values and assumptions of a given environment.

## Preconditions

De Bosscher and colleagues (43) have pointed to the importance of key preconditions including: competent staff who have opportunities for personal and professional development; access to appropriate facilities; quality competition; sufficient funding to support athletes to train as needed. Coach and manager competence appear to be important preconditions for successful HPSEs. Specifically, good managers and coaches have context specific expertise and a clear understanding of their role requirements (44). This supports an even distribution of responsibility and an integration of efforts between domains. Research further suggests that environments should provide coaches with opportunities for personal and professional development and encourage them to improve their hard and soft skills and apply them to the unique environment they are working within (43).

Access to international competition, appropriate facilities, and sufficient funding to support athletes to train as required appear to be important preconditions for a HPSEs success (43, 45). A good structure for national and international level competition opportunities is crucial, where competition should span diverse settings and environments (e.g., changing settings, terrains, different weather conditions etc., (46). Elite athletes must also have access to appropriate facilities that are of a quality that can prepare them for competition and have access to any specialized equipment that is required at the elite level. Similarly, financial resources must be available to allow athletes to train as required (43).

## Processes

Processes reflecting successful HPSEs include a focus on balance in training and recovery, encompassing support, and a holistic approach to coaching. Research suggests that a good environment focuses on monitoring training load, understanding athletes physiological and psychological needs, and adapting training and recovery schedules accordingly (47). 

A focus on holistic support provides a foundation for a good coach-athlete relationship which is an important component of good HPSEs. This relationship is nurtured through a commitment to the athlete's long-term development, a sense of closeness and care between coach and athlete, and a commitment to a collaborative working relationship (48). Researchers point to a strong coach-athlete relationship as being a protective factor against illbeing (49, 50) and dropout (51) and a facilitative factor in wellbeing and performance in elite athletes (48, 52, 53).

HPSEs that adopt a holistic support approach appear to prioritise the development of strong interpersonal relationships among athletes and between athletes and their support staff and coaches, and focus on supporting the whole person, not just the “athlete” (54, 55). The importance of peer friendships is impressed on athletes, and athletes are encouraged to be sources of support for one another in dealing with concerns related to both their sporting and personal lives, despite often being in-competition with each other. Similarly, coaches and support staff focus on developing meaningful and trusting relationships with athletes and coaches and support athletes both in and out of sport (56, 57). Research has illustrated that supportive relationships protect athletes' psychological health, and help athletes develop resilience and enable performance (58, 59).

## Organisational culture

The organisational culture of HPSEs appears to play an important role in their sustainability and success (60, 61). While culture as a construct has been a subject of debate current literature suggests that a facilitative culture in a HPSE is characterised by psychological safety, autonomy, and excellence (62). A psychologically safe environment is one in which there is a “shared belief held by members of a team or environment that the environment is safe for interpersonal risk-taking” (61 p.350). In sport, a psychologically safe environment is reflected by open communication between members, and an openness to seek feedback, propose new ideas and discuss errors without fear of ridicule, rejection, or punishment (63).

Research suggests that a culture characterised by ownership, empowerment and expectations of excellence reflects a good elite sport culture (43, 64–66). A culture of ownership, empowerment and excellence is developed by (1) involving athletes in the development of shared values and goals (2) encouraging athletes to take ownership of their decisions and their development (3) setting high performance expectations and encouraging athletes to be strive for excellence in and out of sport (65, 67). Through promoting autonomy and ownership, the culture itself is strengthened, along with athletes' investment in nurturing it (64).

Taken together, literature points to potential common features across HPSEs that contribute to their ability to support athletes' performance and wellbeing.

## Discussion 

Elite sport research conducted from a psychological vantage point has long sought to understand the determinants of expert performance. This research has had multiple foci, including the traits and skills of the individual athlete (68) characteristics of national programs and policies (31), and aspects of the sport environment (5, 57). While this literature collectively informs practitioners' work in providing an optimal performance setting for their elite and professional athletes, no research has to date made the HPSE the central object of investigation.

In the present paper we suggest that an investigation of the HPSE could be situated between several intersecting discourses that are currently focused on research seeking to understand the role of the environment, and which speak to a more holistic approach to athlete performance. To conceptualise the HPSE, we draw on ideas presented in current athlete career and transition discourse, most notably to understand the successful environment as one that supports career excellence which encapsulates wellbeing, career, and performance dimensions, rather than purely performance. This aligns with consensus thought in the athlete mental health discourse that a win-at-all-costs approach to elite sport is detrimental for athlete wellbeing and may contribute to poor performance outcomes. Research from the applied elite sport psychology discourse highlights the unique pressures inherent to a career at the elite level, which, together with research in elite sport policy discourse, illustrates that HPSEs are unique and exist within a distinct context, yet they exhibit common characteristics attributable to their shared orientation towards performance excellence. Lastly, the HEA discourse calls into focus the idea of the sport environment as a distinct entity that should be studied accordingly.

While we have provided a suggestion above, the HPSE as a distinct phenomenon remains to be defined. While any environment will consist of facilities, people, processes, customs, and additional components, we have in the present paper suggested a definition of an HPSE as a dynamic system. At the micro level, the key focus is an athletes' immediate sport team, but this level also includes their non-sport daily surroundings (e.g., family and friends). These microenvironments are interconnected and situated in a larger macrolevel context that may contain, for example, the national Olympic committee and league policies. Finally, a core component of the HPSE is organisational culture and leadership, which is an integrative factor in supporting athlete performance and wellbeing. The proposed definition requires empirical validation, where focus must be directed towards examining the definition's utility across different HPSEs.

Research across relevant discourses can collectively point to features that may reflect characteristics of successful HPSEs. However, to date, these characteristics have been examined in isolation, the result of which is a body of research that is able only to provide fragmented insights into the interrelationships between the many components of an HPSE, and their relationship to performance and wellbeing. As a result, it remains unclear how factors interact and manifest in practice, and how these factors might work cohesively to influence the success of an HPSE. 

## Future directions 

An integrated understanding of how the key features outlined previously interact and are enacted within an environment is required. This need is in line with discussion calling for a holistic ecological approach in elite sport (25) which would enable a comparison with talent development and provide an understanding of how to support talented athletes in their transition to the elite level. Accordingly, this study underscores key areas for investigation and proposes several prospective research trajectories.

Firstly, future research should consider exploring the perspectives of essential staff through in-depth interviews and observational studies. How do stakeholders and essential staff operating in HPSEs perceive and define success, and how do they wish this success to be operationalised and evaluated? What factors do staff across different environments perceive to be key success factors? And what strategies do individual staff employ in their environments? Answering such questions would help elucidate how best to structure and operate environments in a context-sensitive manner and help to refine the typology of HPSEs. In-depth interview studies with coaches and other central staff from different HPSEs would shed light on recurrent behaviours and strategies emblematic of success across contexts.

Secondly, a case study design would support an understanding of the phenomena in context. Inspired by research in ATDE environments, we would suggest that a successful case, a typical case, and a paradigmatic case be selected. The successful case should be selected based on its track record of helping athletes perform and maintain wellbeing while embedded in their environment. A single case study would encompass multiple data. In-depth interviews could be conducted with a diverse set of personnel including sport psychologists, performance directors, managers, coaches, and athletes. Observations could be conducted in multiple settings including training camps, competitions, social activities, and meetings. A case study approach could provide an integrated perspective on the unique roles of staff and their collaborative efforts in cultivating a successful environment and offer an illustrative understanding of how characteristics that support a successful environment are enacted in practice. A multi-case study reflecting the HPSE typology would allow for a better understanding of the unique characteristics of each type of environment and the similarities and differences between them.

Questionnaire or survey instruments would also be valuable for quantifying psychological and social constructs (e.g., motivational climate, interpersonal relationships), allowing for mixed method approaches that integrate both qualitative and quantitative perspectives. Expanding methodological breadth in this way would enable researchers to examine how HPSEs influence performance, mental health and social dynamics across multiple levels of analysis, ultimately providing a more comprehensive understanding of the dynamic nature of HPSEs.

Together these approaches would support the development of a framework for successful HPSEs which would be of value to practitioners and stakeholders. Such a framework would support those working in such environments to observe areas of strength and weakness in their environment and serve as a guide for making practical changes.

## Research challenges 

There are several conceptual and practical challenges unique to studying HPSEs that may inhibit research progress. On a conceptual level, there is a need for consensus on how the “HPSE” should be defined, delineated, and operationalised. Much research purporting to use elite samples has been conducted on adolescent athletes, who may better reflect the talent development level. Additionally, a unified definition of “success” in elite sport, aligning with prevailing discourses on athlete mental wellbeing and sport psychology, is essential.

Several practical challenges make research in elite sport difficult. Gaining access to HPSEs can be difficult; elite performance requires optimal conditions, and changes to an athlete's environment may negatively impact an athlete by becoming an added source of pressure or discomfort. Moreover, athletes and staff may be reluctant to freely share with researchers for fear of exposing “trade secrets” and losing a competitive advantage. The transient nature of elite sport settings further complicates research. Often, strategies implemented to enhance and maintain the environment are short-lived, not lasting long enough to yield discernible changes or demonstrable successes.

Lastly, HPSEs may vary significantly across cultures, shaped by differences in governance models, funding structures, and athlete support systems. Much of the existing literature focuses on Western, Educated, Industrialised, Rich, and Democratic (WEIRD) contexts, potentially overlooking key differences in non-WEIRD settings. These cultural and contextual variations influence how HPSEs are structured, managed, and resourced. To develop a more comprehensive understanding of HPSEs, future research should incorporate studies from non-WEIRD contexts, capturing the unique cultural nuances that define elite sport environments worldwide.

## Conclusion 

This paper aimed to lay the foundation for future research by providing a contextually situated definition and taxonomy of HPSEs and pointing to key features that should be included in future empirical studies of HPSEs. Our understanding of HPSEs and elite performance is currently limited by a lack of integration and coherence in the research. Research exploring individual characteristics of high-performance and HPSE has provided a valuable foundation, and it is now time for ecological perspectives to further and integrated understanding in this area.

Guided by the HEA and the concept of career excellence, a future mapping of what constitutes a successful HPSE has the potential to help practitioners in such environments realise the strengths and weaknesses of their environments and improve them accordingly.
